# Cholangiocarcinoma diagnosis and treatment

**DOI:** 10.2478/abm-2024-0007

**Published:** 2024-04-30

**Authors:** Editorial Office of Asian Biomedicine

**Affiliations:** Faculty of Medicine, Chulalongkorn University, Bangkok 10330, Thailand

Cholangiocarcinoma (CCA) is a tumor of the bile duct epithelium. It can be categorized into perihilar, intrahepatic, and extrahepatic subtypes depending on its anatomical location [1]. Cancers of the gall bladder or ampulla of Vater can cause similar presentation but they are not classified as CCA.

Early diagnosis is difficult because most patients only have vague symptoms and may not seek care early enough. Most CCA patients present with asymptomatic jaundice, vague right upper quadrant abdominal discomfort, and some weight loss. Some patients, particularly those with intrahepatic CCA, may not have jaundice until late in the natural history of the disease [2].

Patients with asymptomatic jaundice, vague abdominal discomfort, and some weight loss should be properly investigated [3]. Early diagnosis may be documented with various imaging modalities, such as transabdominal ultrasound, computed tomography, and magnetic resonance imaging. These imaging modalities have been used to guide biopsies and CCA staging. Characteristically, extrahepatic CCA shows abrupt changes in ductal diameter with upstream ductal dilation. Endoscopic ultrasound (EUS) and endoscopic retrograde cholangiopancreatography (ERCP) are subsequently used for the evaluation of extrahepatic CCA. Tissue is obtained through EUS fine-needle aspiration or ERCP (biopsy and brush cytology), and therapeutic intervention (such as stent insertion) is performed with ERCP [3]. Lymph node biopsy may help confirm the diagnosis.

All patients with suspected CCA should be investigated for specific tumor markers (carbohydrate antigen) 19-9 or CA 19-9, and carcinoembryonic antigen (CEA) [4]. Patients with intrahepatic lesions should also be checked for alpha-fetoprotein (AFP) [4]. Elevated tumor markers may help support a diagnosis of CCA. An elevated AFP suggests a diagnosis of hepatocellular carcinoma. Recently, 68Ga-FAPI-46 demonstrated superior radiotracer uptake in lesion detection in patients with CCA. The value of immunohistochemistry in the clinical diagnosis of CCA needs further investigation [5].

Recently, it has been suggested that most risk factors for CCA result in chronic inflammation and cholestasis. Chronic inflammation and cholestasis may activate intracellular pathways causing reactive cell proliferation, mutations, and cholangiocarcinogenesis [6]. These changes may help identify bio-markers for clinical diagnosis [6]. In addition to diagnosis, these tumor markers may be used to monitor the status of patients once specific treatment such as surgery has been initiated.

In the present issue, Lu and Li [7] reported the “Treatment of CCA by pGCsiRNA-vascular endothelial growth factor (VEGF) *in vivo.*” They investigated whether pGCsiRNA-VEGF can affect the onset and progression of CCA. They reported that the tumor volume and the weight of pGCsiRNA-VEGF group were significantly smaller than those of mock and si-scramble groups (*P* < 0.05). The expressions of VEGF, matrix metalloproteinases-2 (MMP-2), and MMP-9 at the transcriptional and translational levels were inhibited by pGCsiRNA-VEGF. pGCsiRNA-VEGF promoted tissue apoptosis and destroyed the tissue structure. They concluded that the silencing of VEGF can affect cell survival, and inhibit cell migration, invasion, and development, probably by enhancing apoptosis and inhibiting the expressions of MMP-2 and MMP-9 [7].

Further researches in experimental animals and clinical studies are needed to see whether VEGF can be included in our armamentarium for early diagnosis of CCA. Liver transplants can be offered to patients with very early intrahepatic CCA [8]. Chemotherapy, targeted therapy, and immune checkpoint inhibitors have been used in more advanced stages of the disease [8].
